# First report of *Xiphinema brevicolle* Lordello et Costa, 1961 (Nematoda, Longidoridae) in Japan

**DOI:** 10.3897/zookeys.135.1716

**Published:** 2011-10-07

**Authors:** Hiromichi Sakai, Ai Takeda, Takayuki Mizukubo

**Affiliations:** 1 National Agriculture and Food Research Organization, Agricultural Research Center, Kannondai 3-1-1, Tsukuba, Ibaraki 305-8666, Japan; 2 Chiba Prefectural Agriculture and Forestry Research Center, 808 Daizenno, Midori-Ku, Chiba 266-0006 Japan

**Keywords:** COI, *Ilex crenata*, Japanese holly, rDNA, *Xiphinema americanum*-group, *Xiphinema brevicolle*-subgroup

## Abstract

Mixed populations of *Xiphinema americanum*-group species were detected from a root zone soil sample of Japanese holly, *Ilex crenata*, during a survey for plant-parasitic nematodes of commercial ornamental plant nurseries in Chiba Prefecture, Japan. From the result of the morphological study, the species were identified as *Xiphinema brevicolle* and *Xiphinema* sp. This is the first record of *Xiphinema brevicolle* in Japan. Morphometrics of *Xiphinema brevicolle* generally agree with those of the type specimens and the topotype specimens. *Xiphinema* sp. morphometrically resembles *Xiphinema paramonovi* except for tail length. The mitochondrial COI region, the nuclear 18S rDNA and the nuclear large subunit rDNA D2/D3 region of the species were sequenced and compared in the molecular study. For the COI region, PCR primers were newly designed to obtain longer sequences, ca. 900 bp, than previously used. Sequence identities of COI, 18S and D2/D3 regions between these two populations were 84.0-84.1%, 99.9% and 98.1-98.2%, respectively. Phylogenetic analyses of maximum likelihood trees were carried out to compare genetic relationships among the group and some suggestions were made on the *Xiphinema brevicolle*-subgroup.

## Introduction

During a survey for plant-parasitic nematodes of commercial ornamental plant nurseries in Chiba Prefecture, Japan, we detected mixed populations of *Xiphinema americanum*-group species from a root zone soil sample of Japanese holly, *Ilex crenata* Thunb., one of the major garden tree species in Japan. This study was conducted using morphological characters of females and DNA sequences of the mitochondrial COI region and the nuclear ribosomal RNA (rDNA) regions to identify and characterize the species in the mixed populations.

## Methods

### Collection of the nematode specimens

The soil sample containing the *Xiphinema* spp. (No. 001-001) was taken from the root zone of Japanese holly growing in a commercial plant nursery at Sosa City, Chiba, Japan. Nematodes were extracted with Cobb’s wet-sieving technique. Material collected on a 75 µm mesh sieve was placed on a Baermann funnel and nematodes were collected after one day at room temperature.

### Morphological observation

Females of *Xiphinema* spp. were removed from the nematode suspension using a dissecting microscope and nematode pick. Female nematodes were transferred to a small amount of water then killed by either heating at 60°C for 2 min or by adding hot FP4:1 (Netscher and Seinhorst 1969). Killed nematodes were fixed with FG4:1 (De Grisse 1969). The specimens were fixed for more than one week, processed into glycerin by the ethanol/glycerin method (Seinhorst 1959, De Grisse 1969), and mounted in dehydrated glycerin supported with both minute glass beads and paraffin on glass slides. Morphological observations were made using a DIC microscope (BX51, Olympus Co., Japan). A digital camera, Olympus DP20 or DP21 was used for measuring and taking photo images. The body length and position of vulva were measured with a digital curvimeter (CV-9 Jr., Koizumi Sokki Mfg. Co. Ltd., Japan) on nematode line drawings prepared using a drawing tube. Illustrations of nematodes were sketched directly on highly transparent tracing film (No. 200Z-A4 (S): Tochiman Technical Paper Co. Ltd., Japan) by tracing DP21 digital camera images on a panel-protected LC-display. Sketched images were converted to digital images using the software Adobe Illustrator CS4 (Adobe Systems Inc., USA) and a pen tablet (Intuos4: Wacom Co., Ltd., Japan).

### Molecular study

DNA extractions from single female specimens were carried out according to Sakai (2010), which yielded 200 μL lysate for each specimen. Before DNA extraction, the specimens were tagged, killed by gentle heat, prepared as temporary water mounts, digital images of mounts were photographed, and then images were measured for body and odontostyle length, respectively.

DNA fragments of the mitochondrial COI region were amplified by PCR using the set of primers, CO1-F1 and CO1-R1 (Table 1), which were originally designed from sequence comparison of the COI region between *Xiphinema americanum* (He et al. 2005a: GenBank accession AY382608; NCBI Reference Sequence NC_005928) and *Caenorhabditis elegans* (Howe and Denver 2008: GenBank accession EU407804). CO1-R1 is virtually identical to COIR (He et al. 2005a), with the latter having only a difference in degeneracy. The PCR reaction mixture consisted of 0.2 mM dNTPs, 0.3 μM of each primer, 0.5 U PrimeSTAR HS DNA Polymerase with PrimeSTAR Buffer (5 mM Mg_2_^+^ plus) (Takara Bio Inc., Japan), and 10 μL of DNA lysate as PCR template, in a total volume of 20 μL. The reaction conditions were as follows: a single step of pre-denaturation at 94°C for 2 min, followed by 35 cycles of denaturation at 94°C for 30 s, annealing at 40°C for 15 s, 0.5°C/s ramp up to extension temperature, and extension at 72°C for 1 min.

DNA fragments of the nuclear 18S rDNA region were amplified using the set of primers, 18S 39 and 18S 1573R (Mullin et al. 2005). The PCR reaction mixture consisted of 0.2 mM dNTPs, 0.2 μM of each primer, 0.5 U TaKaRa Ex Taq Hot Start Version with Ex Taq Buffer (Mg_2_^+^ plus) (Takara Bio), and 2 μL of DNA lysate as PCR template, in a total volume of 20 μL. The reaction conditions were as follows: a single step of pre-denaturation at 94°C for 2 min, followed by 35 cycles of denaturation at 94°C for 30s, annealing at 55°C for 30s, and extension at 72°C for 1 min.

DNA fragments of the nuclear large subunit rDNA D2/D3 region were amplified using the set of primers, D2Ab (De Ley et al. 1999) and D3B (Thomas et al. 1997). The PCR reaction conditions were the same as those for 18S rDNA.

These PCR products were purified with QIAquick PCR Purification Kit (Qiagen K.K., Japan), subjected to cycle sequencing with BigDye Terminator v3.1 Cycle Sequencing Kit (Life Technologies Japan), purified with either DyeEx 2.0 Spin Kit (Qiagen) or Agencourt CleanSEQ (Beckman Coulter Inc., USA), and sequenced on the ABI PRISM 3100 Genetic Analyzer (Life Technologies Japan Ltd., Japan). Primers 18S 599R, 18S 550, 18S 977R, 18S 965 (Mullin et al. 2005), and D3B (Thomas et al. 1997) were also used as inner primers for sequencing as well as those designed by us (Table 1). The DNA sequences were aligned by MUSCLE (Edgar 2004) and arranged using BioEdit (Hall 1999). Multiple alignments were manually refined where necessary.

Phylogenetic analyses including substitution model selections were performed using MEGA5 (Tamura et al. 2011) to compare the obtained sequences with homologous sequences of the *Xiphinema americanum*-group in GenBank searched via BLAST. A maximum likelihood (ML) tree was constructed using 1,578 sites for multiply aligned sequences of the 18S rDNA region, where the model K2 + G, the heuristic search with the Close-Neighbor-Interchange (CNI) method, bootstrapping with 500 replications, gaps treatment using all sites, and the neighbor-joining (NJ) tree as the initial tree were employed. A ML tree was constructed using 676 sites for multiply aligned sequences of the D2/D3 region with gaps and an ambiguous site deleted, where the model K2 + G, the CNI method, bootstrapping with 500 replications, and a maximum parsimony (MP) tree as the initial tree were employed. A ML tree was constructed using 342 sites for multiply aligned sequences of the COI region, where the model T92 + G + I, the CNI method, bootstrapping with 500 replications, and a MP tree as the initial tree were employed. Among COI homologous sequences of the *Xiphinema americanum*-group in the database, those including alignment gaps were excluded from the analysis because they possibly represent pseudogenes. Sequences of *Longidorus* species available in GenBank were used to root those trees.

**Table 1.** Primers designed and employed originally in this study for PCR amplification and sequencing of mitochondrial COI region and rDNA D2/D3 region.

primer name	Sequence	Note
CO1-F1	5’-ATAATTTTTTTTATGGTAATACC-3’	PCR, sequencing
CO1-R1	5’-ACTACATAATAAGTATCATG-3’	PCR, sequencing
CO1-F2	5’-TATATTTTAATTTTACCTGG-3’	sequencing
CO1-R2	5’-CCAGGTAAAATTAAAATATA-3’	sequencing
D3A-R	5’-AGACTCCTTGGTCCGTGTTTC-3’	sequencing

## Results

The DNA sequence analysis of the mitochondrial COI region for 9 specimens suggested two different *Xiphinema americanum*-group species were present. Morphometric data were used to identify them as *Xiphinema brevicolle* Lordello et Costa, 1961, and an undescribed *Xiphinema americanum*-group species.

### Morphological observations

#### Xiphinema brevicolle

Lordello & Costa, 1961

http://species-id.net/wiki/Xiphinema_brevicolle

**Figure 1.**
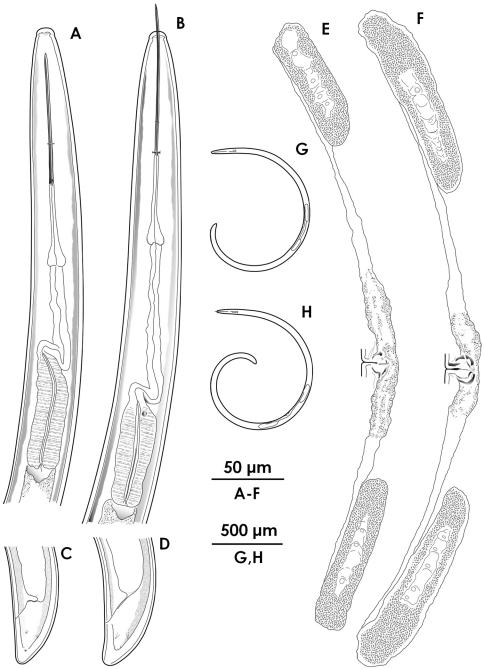
Female morphology of *Xiphinema brevicolle* **(A, C, E, G)** and *Xiphinema* sp. **(B, D, F, H) A–B** Anterior region **C–D** Tail **E–F** Reproductive system **G–H** Entire body.

Figs 1A, 1C, 1E, 1G 2 4A 5A 6A

##### Measurements.

See Tables 2, 3.

##### Remarks.

Morphometrics of the specimens obtained here generally agree with those of the type specimens and topotype specimens (Lamberti et al. 1992, Luc et al. 1998) of the species (Table 2). No male was detected.

##### Nomenclatorial note.

The emended name of this species, *Xiphinema brevicollum*, was proposed by Luc et al. (1998) and used in many works to date. Monteiro (2010) claimed that the correct species name is *Xiphinema brevicolle* Lordello et Costa, 1961, and should have been preserved unaltered. We support the claim by Monteiro (2010) and *Xiphinema brevicolle* is used here.

#### Xiphinema sp.

Figs 1B, 1D, 1F, 1H 3 4B 5B 6B

##### Measurements.

See Tables 2, 3.

##### Remarks.

These specimens morphometrically resemble *Xiphinema paramonovi* Romanenko, 1981, except for the clearly different tail length (Table 2). The morphometrics of the specimens partly overlap those of *Xiphinema brevicolle*. General morphology and DNA information addressed below suggest that these specimens belong to some species related to *Xiphinema brevicolle*, though a specific species accommodating them was not found. We finally regarded these specimens as an unidentified *Xiphinema americanum*-group species. Further information is required to identify the specimens as a new species or determine if they represent intra-specific variation of species previously described. No male was detected.

**Table 2.** Morphometrics of *Xiphinema brevicolle*, *Xiphinema* sp., *Xiphinema paramonovi* and *Xiphinema diffusum* (female). Mean ± standard deviation (range) in μm, except for L in mm, and ratios.

	*Xiphinema brevicolle*				*Xiphinema* sp.(this study)	*Xiphinema paramonovi* Paratypes(Romanenko 1981)	*Xiphinema diffusum* Paratypes (Lamberti and Bleve-Zacheo 1979)
	Populationin this study	Topotypes		Types (Lordello and Costa 1961)			
		(Luc et al. 1998)	(Lamberti et al. 1992)				
n	22	25	17	-	13	27	10
L	1.93 ± 0.11(1.71-2.10)	1.92 ± 0.122(1.7-2.16)	2.1 ± 0.1(1.8-2.2)	(1.82-2.20)	2.30 ± 0.12(2.08-2.47)	2.1(2.0-2.3)	1.7(1.6-1.8)
a	46.1 ± 2.6(40.5-50.5)	46.1 ± 1.72(44-51)	44.5 ± 2.3(40.7-50.1)	(36.0-42.2)	48.8 ± 2.4(45.0-52.3)	49.6(44-54.2)	47(46-51)
b	6.6 ± 0.6(5.5-8.2)	5.92 ± 0.37(5.1-6.7)	6.4 ± 0.6(5.6-7.7)	(7.0-10.5)	7.0 ± 0.4(6.5-8.1)	6.1(4.8-7.1)	6.9(5.3-8.9)
c	69.8 ± 4.4(60.5-79.1)	76.9 ± 5.64(67.6-89.9)	77.8 ± 0.6(60.3-94.0)	(62.5-93.0)	79.6 ± 8.5(67.5-94.5)	60.5(49.1-68.5)	72(63-84)
c’	1.0 ± 0.1(0.9-1.2)	0.96 ± 0.06(0.89-1.10)	1.0 ± 0.06(0.9-1.1)	-	0.9 ± 0.1(0.8-1.2)	1.1(0.9-1.2)	0.9(0.8-1.1
V	50.5 ± 1.2(48.1-53.6)	53 ± 1.9(50-55)	53 ± 0.9(51.0-54.0)	(50.0-54.0)	52.4 ± 1.1(50.9-54.0)	52.1(50.8-55.0)	50(47-52)
Total stylet	149.0 ± 4.4(144-161)	159 ± 8.05(144-173)	-	(156.0-168.3)	166.5 ± 2.6(161-171)	-	-
Odontostyle	94.1 ± 3.3(88-102)	101 ± 6.14(89-110)	101.9 ± 7.2(84.7-108.2)	-	107.3 ± 2.7(103-111)	103.5(88.5-120.0)	87(84-89)
Odontophore	54.9 ± 2.1(51-59)	59 ± 3.43(50-64)	57.0 ± 2.9(48.8-60.0)	(61.2-62.7)	59.2 ± 1.6(57-62)	56.7(53.1-60.0)	50(48-51)
Oral apertureto guide ring	76.5 ± 3.8(67-83)	86.0 ± 4.23(77-92)	86.3 ± 5.6(72.3-92.3)	-	85.5 ± 5.0(78-93)	79.6(66.0-103.0)	62(60-64)
Pharyngeal bulblength	79.3 ± 4.0(73-86; n = 10)	-	-	(61-79)	84.9 ± 2.7(82-90; n = 7)	94.2(65.0-117)	-
Pharyngeal bulbwidth	21.2 ± 1.2(18-23; n = 21)	-	-	(12-19)	22.9 ± 1.7(20-26)	21.1(18.0-24.0)	-
Tail	27.8 ± 2.0(25-32)	26.0 ± 1.71(23-28)	26.8 ± 2.0(24.1-31.2)	(24.5-29.0)	29.2 ± 2.8(24-34)	36.1(33.0-47.0)	24(21-28)
Hyaline portionof tail (J)	10.4 ± 1.5(8-13)	-	8.0 ±0.9(5.9-9.4)	-	11.4 ± 1.5(9-14)	9(7-11)	12(10-14)
Body diam.at lip region	12.5 ± 0.4(12-13)	11.5(11-13)	11.5 ± 0.5(10.6-12.3)	-	12.4 ± 0.4(11-13)	14.6(13.5-15.0)	11(10-12)
Body diam.at guide ring	30.4 ± 0.8(29-32)	-	29.8 ± 1.5(27.1-31.8)	-	32.6 ± 0.8(31-34)	31.7(30.0-36.0)	26(26-27)
Body diam.at base of pharynx	37.9 ± 1.2(35-40)	-	39.4 ± 2.9(35.3-45.3)	-	41.3 ± 2.2(38-45)	40.1(36.0-42.0)	33(31-35)
Body diam.at vulva	42.0 ± 2.0(38-46)	-	46.6 ± 3.4(39.4-50.0)	(49.0-59.7)	47.2 ± 2.5(44-52)	43.4(39.0-47.2)	36(33-38)
Body diam.at anus	27.0 ± 1.6(24-30)	26(22-29)	26.6 ± 1.7(21.8-29.4)	(30.6-36.7)	29.7 ± 1.1(28-32)	32.4(27.0-41.0)	25(23-28)
Body diam.at beginning of J	16.2 ± 2.2(12-20)	-	13.7 ± 1.1(11.2-14.7)	-	18.4 ± 2.2(15-22)	7(6-8)	17(15-20)

**Table 3.** Uterine and vaginal region lengths of *Xiphinema brevicolle* and *Xiphinema* sp. measured in this study (female). Mean ± standard deviation (range; number of specimens) in μm, except where indicated.

	*Xiphinema brevicolle*	*Xiphinema* sp.
Anterior uterus length	44.3 ± 4.8(34-54; n = 14)	35.1 ± 2.9(32-40; n = 9)
Posterior uterus length	41.1 ± 2.9(37-46; n = 9)	32.7 ± 2.7(29-37; n = 9)
Pars proximalis vaginae	5.9 ± 0.7(5-7; n = 22)	7.6 ± 1.5(5-10; n = 13)
Pars distalis vaginae	10.5 ± 0.7(9-12; n = 22)	11.3 ± 1.0(9-13; n = 13)
Vagina length	16.4 ± 1.0(14-18; n = 22)	18.9 ± 1.5(17-22; n = 13)
Vagina length /body diam. at vulva (%)	39.2 ± 3.2(31.3-44.6; n = 22)	40.1 ± 2.9(34.8-44.2; n = 13)

##### Molecular study.

**Figure 7.**
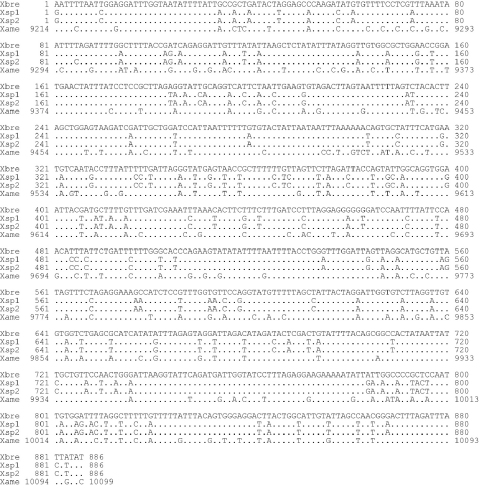
Sequence comparison of mitochondrial COI region. Xbre: *X. brevicolle* (this study: GenBank accession AB604337); Xsp1, Xsp2: *Xiphinema* sp. (this study: AB604338 and AB604339 respectively); Xame: *X. americanum* (He et al. 2005: usAY382608).

DNA sequences of 886 bp except for primer regions were obtained for the mitochondrial COI region. The five *Xiphinema brevicolle* specimens observed had identical sequences, whereas a single nucleotide in the sequence differed among the four specimens of *Xiphinema* sp. though this variation resulted in no difference between the translated amino acid sequences within the specimens. Sequence identity between these two species was 84.0-84.1%, whereas the sequences of *Xiphinema brevicolle* and *Xiphinema* sp. were 80.7 and 80.4-80.5% identical to that of *Xiphinema americanum* (He et al. 2005a) respectively, with no gap found among them (Fig. 7). Putative amino acid sequences were available without any stop codon when translations were made from the second base of the obtained sequences. DNA sequences of 1,566 bp except for primer regions were obtained for the 18S rDNA region from one specimen for each species. The difference between sequences of the two species studied here was only a single nucleotide, resulting in 99.9% identity without any gap. DNA sequences of 788-791 bp except primer regions were obtained for the D2/D3 region from four specimens for each species. A single nucleotide variation of sequence among four specimens of *Xiphinema brevicolle* was observed, whereas no variation of sequence was observed among four specimens of *Xiphinema* sp. Sequence identity between these two species was 98.1-98.2%, with gaps found.

**Figure 8.**
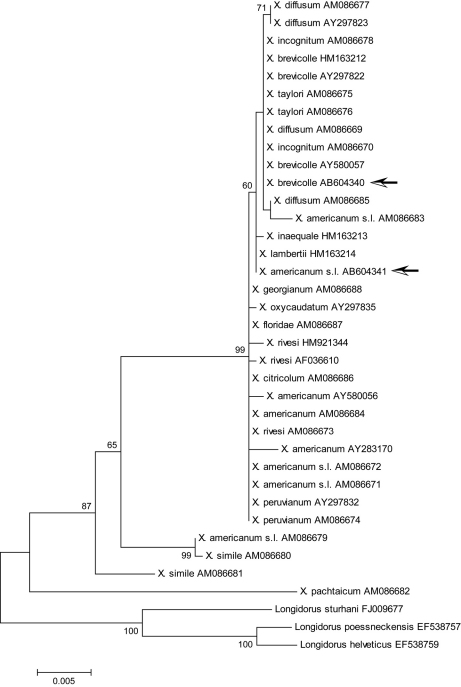
Maximum likelihood tree for 18S rDNA region. Bootstrap values higher than 50 are shown. Arrows indicate specimens examined in this study: *Xiphinema brevicolle* GenBank accession AB604340; *Xiphinema* sp. AB604341. The tree is drawn to scale, with branch lengths measured in the number of substitutions per site.

**Figure 9.**
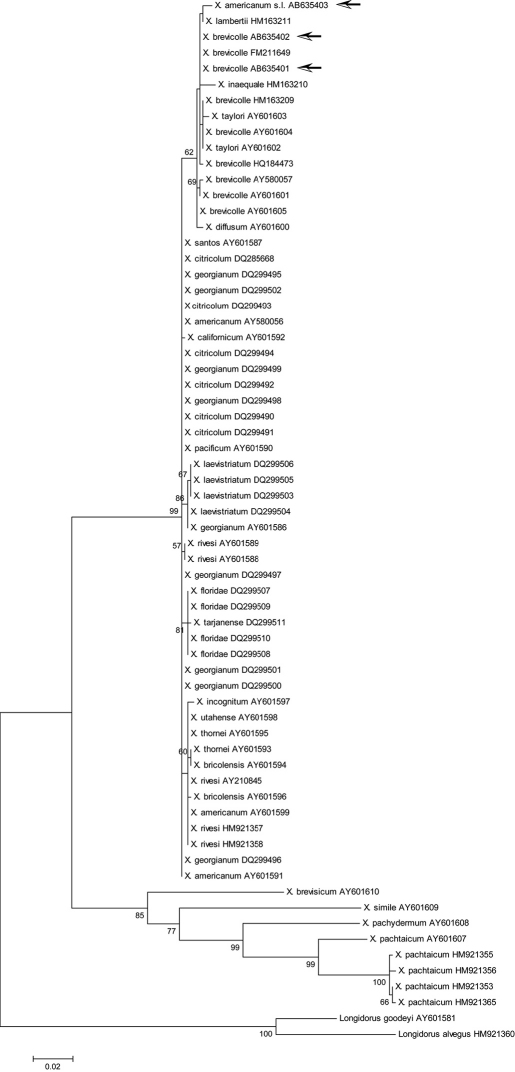
Maximum likelihood tree for D2/D3 region. Bootstrap values higher than 50 are shown. Arrows indicate specimens examined in this study: *Xiphinema brevicolle* GenBank accession AB635401 and AB635402; *Xiphinema* sp. AB635403. The tree is drawn to scale, with branch lengths measured in the number of substitutions per site.

**Figure 10.**
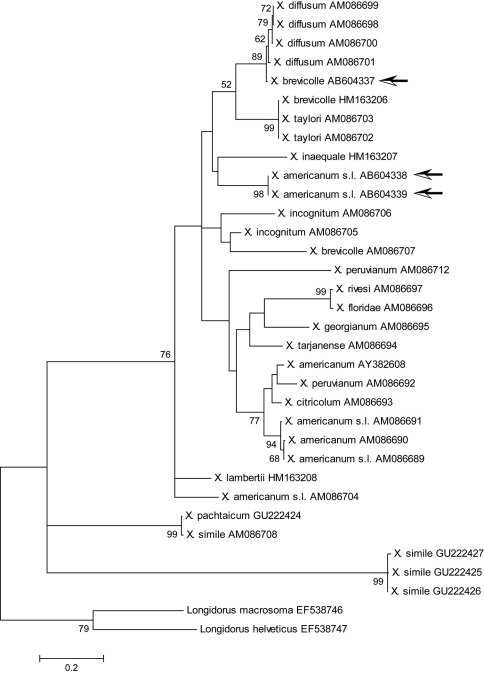
Maximum likelihood tree for COI region. Bootstrap values higher than 50 are shown. Arrows indicate specimens examined in this study. The tree is drawn to scale, with branch lengths measured in the number of substitutions per site.

ML trees inferred for the 18S rDNA and D2/D3 regions placed our specimens in similar clades, which include *Xiphinema brevicolle* and its junior synonyms by Luc et al. (1998) such as *Xiphinema diffusum* Lamberti et Bleve-Zacheo, 1979, *Xiphinema incognitum* Lamberti and Bleve-Zacheo, 1979, *Xiphinema taylori* Lamberti et al., 1992, as well as other different species like *Xiphinema inaequale* (Khan et Ahmad, 1975) and *Xiphinema lamberti* Bajaj et Jairajpuri, 1977 (Figs 8, 9). On the other hand, the ML tree inferred for the COI region didn’t show strong support for such a clade because of a low bootstrap value though several subclades were strongly supported by high bootstrap values (Fig. 10).

## Discussion

This study reports the occurrence of *Xiphinema brevicolle* in Japan for the first time. The only member species of the *Xiphinema americanum*-group recorded to date in Japan was *Xiphinema incognitum* Lamberti & Bleve-Zacheo, 1979, described as a new species for nematode specimens detected from soil of bonsai trees exported from Japan to England (Lamberti and Bleve-Zacheo 1979).

The specimens of this study were obtained from mixed populations of the *Xiphinema americanum*-group. Information on juveniles was not included because the coexistence of the two closely related nematodes requires special care to separate juvenile specimens of the different species. Situations like this will make matters worse if one is to identify the species, since it is difficult enough to identify even a single population of the group in many cases. Detection of mixed populations of *Xiphinema americanum* Cobb, 1913 and *Xiphinema rivesi* Dalmasso, 1969 were reported (Vrain et al. 1992, Vrain 1993), and such coexistence of multiple populations and/or species of the *Xiphinema americanum*-group may be common. Therefore, it is strongly recommended to check the genetic uniformity of a given population to be identified. The mitochondrial COI region has been recently used to examine variations in populations of *Xiphinema* species including members of the *Xiphinema americanum*-group (Lazarova et al. 2006, Kumari et al. 2010a, 2010b), where ca 400 bp sequences were examined. This genetic region has much more information to differentiate between populations than 18S rDNA and D2/D3 regions. As shown above, we developed new primers and examined longer sequences of the COI region than previously used. Examination of longer sequences provides not only more reliable comparative results but also another option to develop a more specific primer to the species to be tested since high variability of this region may contribute to some unfitness of reported primers. Among options available at present, sequences of the COI region can be efficiently used to examine the diversity of *Xiphinema* specimens.

ML phylogenetic analyses of 18S rDNA and D2/D3 regions moderately supported the clade including *Xiphinema brevicolle*, *Xiphinema diffusum*, *Xiphinema phinema inaequale*, *Xiphinema incognitum*, *Xiphinema lamberti*, and *Xiphinema taylori* with our materials (Figs 8, 9), whereas such a clade was not so highly supported in the ML tree of COI (Fig. 10). Member species of *Xiphinema americanum*-group harbor endosymbionts (Vandekerckhove et al. 2000). The difference in phylogenetic inference between nuclear and mitochondrial genetic regions may result from the symbionts’ effect on mitochondrial DNA since unreliable results of phylogenetic inference based on mitochondrial DNA due to the presence of a symbiont are known (Hurst and Jiggins 2005). Furthermore, the ML tree of COI brought another problem to us. It showed closer relationship of our *Xiphinema brevicolle* population to *Xiphinema diffusum* than to other *Xiphinema brevicolle* populations. Our specimens identified as *Xiphinema brevicolle* were reasonably larger than type specimens of *Xiphinema diffusum* (Table 2), and our identification was made considering more overlapping morphometrics of type specimens of *Xiphinema brevicolle*. If COI sequences can differentiate species of *Xiphinema americanum*-group, our specimens may be identified as *Xiphinema diffusum* rather than *Xiphinema brevicolle*. In such a situation, morphological features used to identify species should be reconsidered since a single species like *Xiphinema diffusum* can have a wide range of morphometrics which may result in more difficult diagnoses without reducing the number of species by synonymizing them intensively. In any case, it should be desirable to collect much more COI sequences of other species and populations, such as the sequence data of topotype specimens of *Xiphinema diffusum*, which is unavailable at present. Our results of phylogenetic analyses, however, may be helpful to refine the concept of a *Xiphinema brevicolle*-subgroup, which was previously discussed in some works (Romanenko and Stegaresku 1985, Lamberti and Ciancio 1993, He et al. 2005b). Taking our results of phylogenetic analyses into consideration, we suggest that the *Xiphinema brevicolle*-subgroup includes at least the five species of *Xiphinema brevicolle*, *Xiphinema diffusum*, *Xiphinema inaequale*, *Xiphinema incognitum*,and *Xiphinema taylori*, and our specimens also belong to the subgroup, though the validity of respective species is a different matter. Appropriate establishment of subgroups within the *Xiphinema americanum*-group will contribute to a more feasible identification process of the member species and requires further research.

## Conclusion

In this study, specimens from mixed populations of the *Xiphinema americanum*-group, present in the root zone of Japanese holly in Japan, were identified as *Xiphinema brevicolle* and *Xiphinema* sp. This record of *Xiphinema brevicolle* is the first for Japan. PCR primers to amplify longer sequences of the mitochondrial COI regions were originally designed and used to efficiently differentiate the specimens. Phylogenetic analyses using 18S rDNA, D2/D3, and COI regions supported a close relationship among our specimens and species related to *Xiphinema brevicolle* or the *Xiphinema brevicolle*-subgroup.

## Supplementary Material

XML Treatment for Xiphinema brevicolle

XML Treatment for Xiphinema

## Figures and Tables

**Figure 2.**
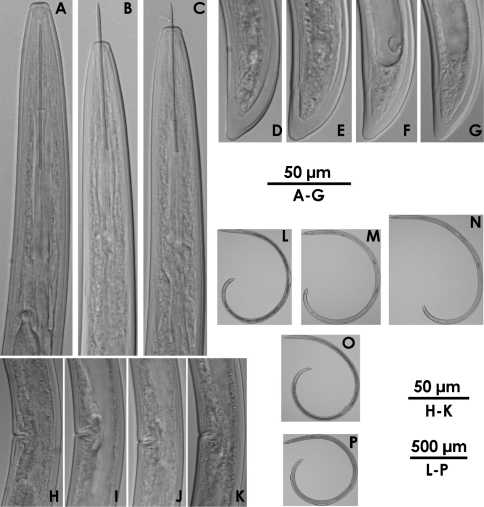
*Xiphinema brevicolle* (female) **A–C** Anterior region **D–G** Tail **H–K** Vulval region **L–P** Entire body.

**Figure 3.**
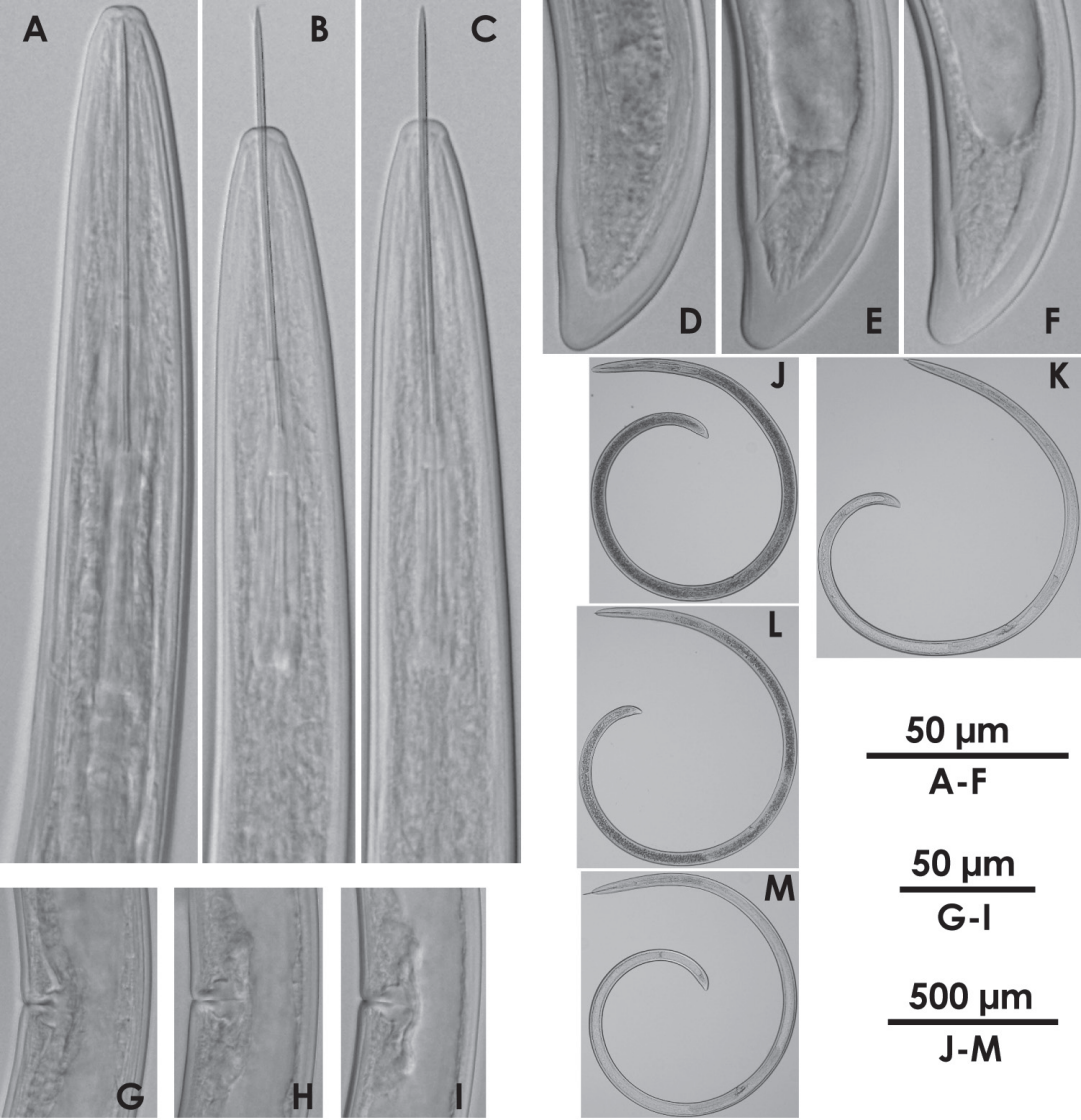
*Xiphinema* sp.(female) **A–C** Anterior region **D–F** Tail **G–I** Vulval region **J–M** Entire body.

**Figure 4.**
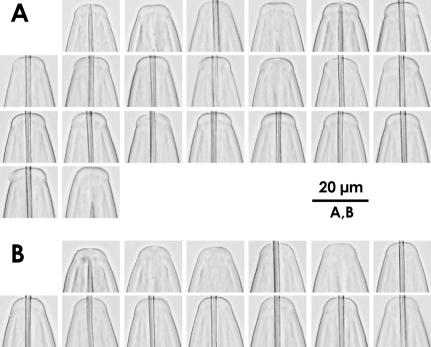
Intra-population variation of lip region in females **A** *Xiphinema brevicolle* **B** *Xiphinema* sp.

**Figure 5.**
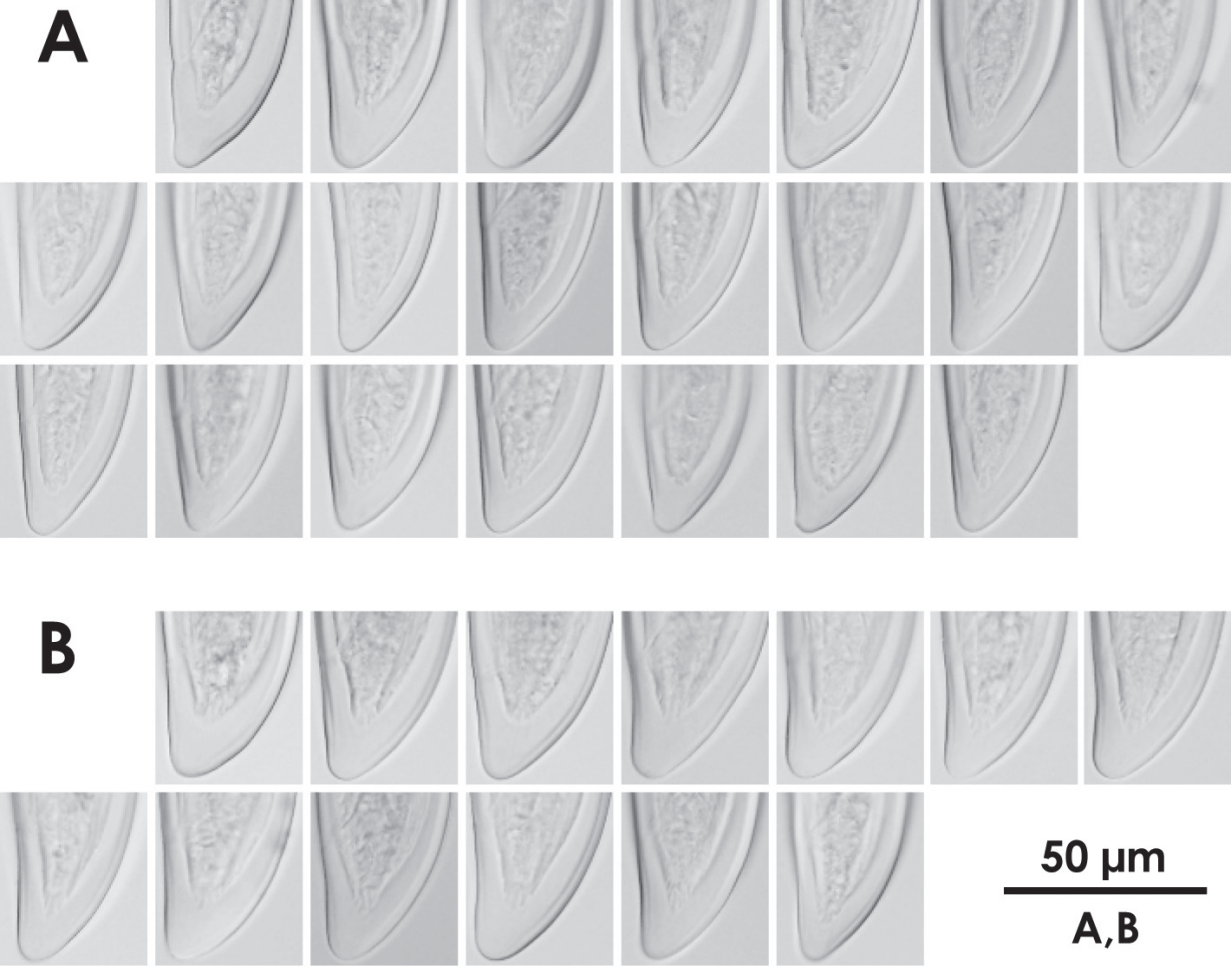
Intra-population variation of tail in females **A** *Xiphinema brevicolle* **B** *Xiphinema* sp.

**Figure 6.**
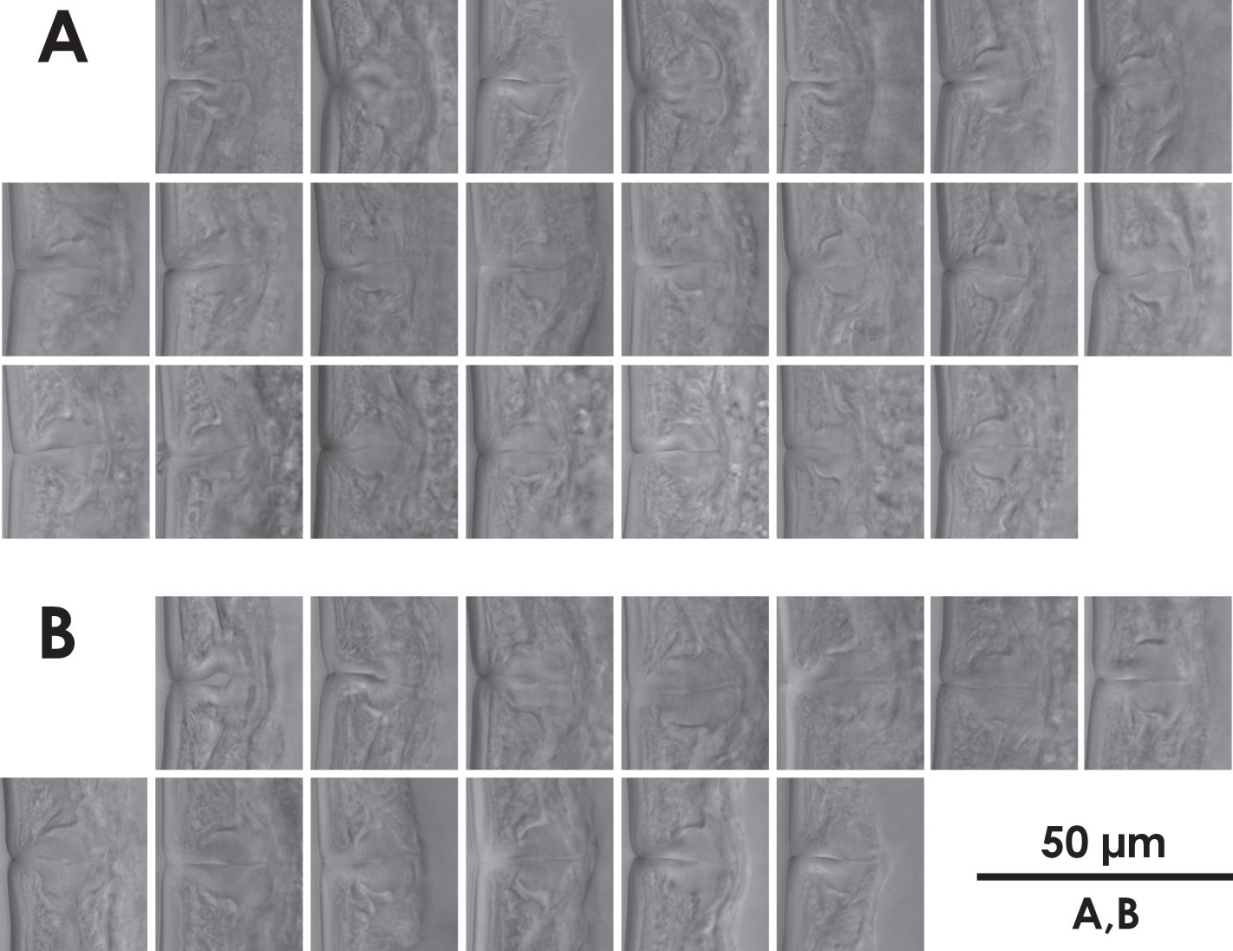
Intra-population variation of vagina in females **A** *Xiphinema brevicolle* **B** *Xiphinema* sp.
